# Autophagy Protects against Eosinophil Cytolysis and Release of DNA

**DOI:** 10.3390/cells11111821

**Published:** 2022-06-02

**Authors:** Stephane Esnault, Paul S. Fichtinger, Karina T. Barretto, Frances J. Fogerty, Ksenija Bernau, Deane F. Mosher, Sameer K. Mathur, Nathan Sandbo, Nizar N. Jarjour

**Affiliations:** 1 Division of Allergy, Pulmonary and Critical Care Medicine, Department of Medicine, University of Wisconsin-Madison School of Medicine and Public Health, Madison, WI 53726, USA; psfichtinger@medicine.wisc.edu (P.S.F.); kbernau@medicine.wisc.edu (K.B.); skmathur@wisc.edu (S.K.M.); nsandbo@medicine.wisc.edu (N.S.); njarjour@uwhealth.org (N.N.J.); 2 Department of Biomolecular Chemistry, University of Wisconsin-Madison School of Medicine and Public Health, Madison, WI 53726, USA; kbarretto@wisc.edu (K.T.B.); ffogerty@medicine.wisc.edu (F.J.F.); dfm1@medicine.wisc.edu (D.F.M.); 3 Division of Hematology and Oncology, Department of Medicine, University of Wisconsin-Madison School of Medicine and Public Health, Madison, WI 53726, USA

**Keywords:** eosinophils, autophagy, cytolysis, DNA traps, SQSTM1, MAP1LC3B, IL3, IL5, IgG

## Abstract

The presence of eosinophils in the airway is associated with asthma severity and risk of exacerbations. Eosinophils deposit their damaging products in airway tissue, likely by degranulation and cytolysis. We previously showed that priming blood eosinophils with IL3 strongly increased their cytolysis on aggregated IgG. Conversely, IL5 priming did not result in significant eosinophil cytolysis in the same condition. Therefore, to identify critical events protecting eosinophils from cell cytolysis, we examined the differential intracellular events between IL5- and IL3-primed eosinophils interacting with IgG. We showed that both IL3 and IL5 priming increased the eosinophil adhesion to IgG, phosphorylation of p38, and production of reactive oxygen species (ROS), and decreased the phosphorylation of cofilin. However, autophagic flux as measured by the quantification of SQSTM1-p62 and lipidated-MAP1L3CB over time on IgG, with or without bafilomycin-A1, was higher in IL5-primed compared to IL3-primed eosinophils. In addition, treatment with bafilomycin-A1, an inhibitor of granule acidification and autophagolysosome formation, enhanced eosinophil cytolysis and DNA trap formation in IL5-primed eosinophils. Therefore, this study suggests that increased autophagy in eosinophils protects from cytolysis and the release of DNA, and thus limits the discharge of damaging intracellular eosinophilic contents.

## 1. Introduction

Eosinophilic diseases such as asthma, chronic rhinosinusitis, eosinophilic gastrointestinal diseases, and hypereosinophilic syndromes display systemic and tissue eosinophilia, which contribute to chronic inflammation, tissue damage, and remodeling [1,2,3].

Several studies have reported the presence of lysed eosinophils, as well as the presence of both free eosinophil granules and extracellular DNA in tissues in several eosinophilic diseases and models [4,5,6,7,8,9,10]. Notably, the presence of free eosinophil granules has been associated with epithelial damage in the airways [11]. The release of eosinophil content during cytolysis is concomitant with extracellular projections of DNA (eosinophil extracellular trap formation) that carries histones, toxic proteins and eosinophil granules [12,13,14], the latter of which can further release toxic proteins and other eosinophilic mediators, causing lasting tissue damage [15]. Therefore, unlike a death by apoptosis, cytolysis is likely to cause more inflammation and tissue damage.

Despite the observations of lysed eosinophils and free granules in tissues, very little has been reported concerning mechanisms of eosinophil cytolysis. In a study by Radonjic-Hoesli et al., cytolysis was induced via IgG interaction and activation with the complement iC3b, which trigger necroptosis upstream of dihydronicotinamide-adenine dinucleotide phosphate (NADPH)-dependent reactive oxygen species (ROS) production [16]. In that study, rapamycin-enhanced autophagy was reported to be protective against eosinophil cytolysis.

We have recently shown that long-term priming of eosinophils with interleukin (IL)3 led to the production of the low-affinity IgG receptor (CD32), activation of both CD32 and the αMβ2 integrin, eosinophil adhesion to IgG via phosphatidylinositol 3′-kinase (type-I PI3K), increased ROS production by NADPH oxidase, change in microtubule dynamics, increased p38 mitogen-activated protein kinases (MAPK)-phosphorylation and reduction in ROCK-mediated phosphorylation of cofilin, all leading to important eosinophil cytolysis when interacting with IgG [17,18]. Conversely, possibly due to a reduction in the specific IL5 α-chain receptor on the eosinophil surface over time [19,20,21], long-term priming with IL5 did not lead to significant eosinophil lysis on IgG [18]. Thus, comparing intracellular events in these two long-term priming models that are only differentiated by the use of two cytokines of the common β-chain receptor family (IL3 and IL5), we aimed to identify specific mechanisms involved in eosinophil cytolysis. The results prompted us to test the hypothesis that downstream of ROS production, autophagy is differentially regulated by IL5 and IL3 priming.

## 2. Methods

### 2.1. Subjects, Eosinophil Preparation and Cultures

Circulating eosinophils were purified from donors with eosinophils >35 per μL of blood. The participants did not use systemic steroids or take topical/inhaled corticosteroids the day of the blood draw. Informed consent was obtained from each subject prior to participation. The studies were approved by the University of Wisconsin-Madison Health Sciences Institutional Review Board (#2013-1570). As previously described [17], eosinophils were purified by negative selection. Eosinophil preparations with purity >98% and viability ~98% were used. Eosinophils were cultured at 1 × 10^6^/mL in complete medium (RPMI 1640 plus 10% fetal bovine serum) with IL3 (2 ng/mL) or IL5 (2 ng/mL) (both cytokines from BD Biosciences [San Jose, CA, USA]) for 20 h, and were then washed and cultured with complete medium (no IL3 or IL5) on coated heat-aggregated immunoglobulins-G (HA-IgG or IgG) or without IgG as previously described [17]. Human serum IgG was from Sigma-Aldrich (St. Louis, MO, USA).

### 2.2. Adhesion Assays

Eosinophil adhesion using Cell Tag 700 was assessed as previously described [18]. Briefly, after 2 h on IgG, cells were fixed with 4% paraformaldehyde (PFA) and permeabilized by adding 0.1% Triton X-100 in PBS. Then, 50 μL/well of Cell Tag 700 diluted 1:500 in blocking buffer was added for 1 h. Imaging was performed using the LI-COR CLx with a 3.5 μm focus offset and scanned at medium quality with 84 μm resolution. Intensity of Cell Tag 700 staining was measured in the 700 nm channel and quantified using Image Studio 5.2 software. For both adhesion assays, all conditions were performed in quadruplicate.

### 2.3. Western Blot

Eosinophils were primed with cytokines for 20 h and were seeded on HA-IgG for the indicated times. Treatment with bafilomycin-A1 (BioViotaca, Liestal, Switzerland) started after priming and 20 min before seeding on IgG. Cells were lysed directly in LaemmLi buffer (10% SDS plus ß-mercaptoethanol), before boiling and loading onto 15 to 10% SDS-polyacrylamide gels. Proteins were transferred into a PVDF membrane. Immunoblotting was performed as previously described [22]. Blots were subsequently incubated with desired primary antibodies: anti-ß-actin from Sigma-Aldrich, anti-phospho-cofilin, anti-SQSTM1 and anti-LC3B from Cell Signaling Technology (Danvers, MA, USA) and anti-phospho-p38 from Genetel Laboratories (Pasadena, TX, USA) (all diluted by 2000-fold in 1× TBS, 0.1% Tween-20 with 5% BSA). Then, the appropriate HRP-conjugated secondary antibodies (Calbiochem, San Diego, CA, USA) were utilized. Immunoreactive bands were visualized using ECL reagents and GE LAS4000 chemiluminescence imager (GE Healthcare, Chicago, IL, USA) and iBright CL1000 (InVitrogen, ThermoFisher Scientifics, Waltham, MA, USA). Bands were quantified using ImageJ (https://imagej.nih.gov/ij/ accessed on 23 April 2018).

### 2.4. Reactive Oxygen Species (ROS) Measurement

As previously described [18], eosinophils were primed with IL3 or IL5 for 19 h in complete medium before addition of dihydrorhodamine (DRH)-123 (5 µM; Molecular Probes, Invitrogen^TM^, Eugene, OR, USA) for one hour. Then, cells were washed and seeded on HA-IgG or without IgG in 96-well plate in quadruplicate. ROS production was measured by fluorescence (485 nm_Ex_/520 nm_Em_) at the indicated timepoints. Fluorescence measured in medium only (no cells) was subtracted from the values shown. As a positive control for ROS production, IL3-primed eosinophils were treated with PMA (100 ng/mL).

### 2.5. Microscopy

To visualize the release of DNA from IL3-primed eosinophils on IgG for 5 h, eosinophils were cultured with IL3 (2 ng/mL) for 20 h, and 500 µL of cells (0.6 × 10^6^/mL in fresh medium with no IL3) were added to acid-washed or poly-L-lysine-coated coverslips with HA-IgG (10 µg/mL; 500 µL/well). After 5 h of incubation at 37 °C, coverslips were fixed, permeabilized, and stained as previously described [18]. Coverslips were incubated with mouse anti-gelsolin clone 2 (BD Transduction Laboratories, San Diego, CA, USA) diluted 1:100 and Alexa Fluor 647-labeled phalloidin (Invitrogen, Waltham, MA, USA) diluted 1:40, followed by staining with Alexa Fluor 488-conjugated anti-mouse IgG (Cell Signaling Technology, Danvers, MA, USA) diluted 1:1000 and 4′,6-diamidino-2-phenylindole (DAPI). Coverslips were mounted on slides and sequential 0.3-μm z-step images were acquired and reconstructed using a Nikon A1R-Si + Confocal (60 × oil objective) and NIS-Elements AR v4.30 software.

### 2.6. Cytolysis Measurement of Eosinophils on HA-IgG

As previously described [18], after 20 h of cytokine priming and 4.5 h on HA-IgG, 20 µL of bis-alanyl-alanyl-phenylanlanyl-rhodamine 110 (R110; CytoTox-Fluor™ Cytotoxicity Assay, Promega, Madison, WI, USA) was added to each well for 30 min to measure the loss of membrane integrity by fluorescence (485 nm_Ex_/520 nm_Em_). For the condition with bafilomycin-A1 treatment, bafilomycin-A1 (1 μM) was added to cells after the priming and 20 min before seeding on coated HA-IgG. The same concentration of vehicle (DMSO) used to prepare bafilomycin-A1 was used as a control. All conditions were performed in triplicate. The value for the IL3IgG condition (IL3 priming on IgG with DMSO) was fixed at 100.

### 2.7. DNA Spreading and Projections

Eosinophils primed with either IL3 or IL5 (both at 2 ng/mL) for 20 h, treated with bafilomycin-A1 (1 μM) or vehicle alone for 20 min, were seeded at 0.5 × 10^6^ cells/mL (300 μL/well) in an eight-well chamber slide (Ibidi, Gräfelfing, Germany) coated with 300 μL of HA-IgG (10 μg/mL). After 4.5 h, cells were washed and fixed with 4% paraformaldehyde for 20 min at room temperature (RT). After three washes with PBS, DAPI was added into wells for 10 min at RT, followed by three washes and the addition of mounting medium (Ibidi). Images were taken using a Nikon Eclipse Ti microscope and its 20× objective. Bright-field (BF) images and images of DAPI fluorescence (200 msec. exposure) were taken using the BF and DAPI settings on the NIS-Elements AR v4.30.02 software. BF images allowed cell counting while DAPI fluorescence was quantified by ImageJ (https://imagej.nih.gov/ij/ accessed on 23 April 2018) (threshold fixed around 6500). For each well, three to four fields (depending on the cell density in the field) were randomly used for a total cell count of ~450 cells. The total area of DAPI fluorescence per cell was calculated, and the number of DNA projections per 100 cells was counted. A projection was defined as a line of DNA with a length >2-fold the length of an intact nucleus.

### 2.8. Statistical Analyses

Statistical analyses were performed using the SigmaPlot 13.0 software package (Systat Software, Inc., Palo Alto, CA, USA). Differences between two groups were analyzed using Student’s paired *t* test or *t* test as indicated. One-way ANOVA was used to compare more than two groups. *p* < 0.05 was considered statistically significant.

## 3. Results

### 3.1. Both IL5 and IL3 Priming Led to Adhesion on IgG, Phosphorylation of p38, Dephosphorylation of Cofilin and to High Production of ROS after Interaction with IgG

**Figure 1:**
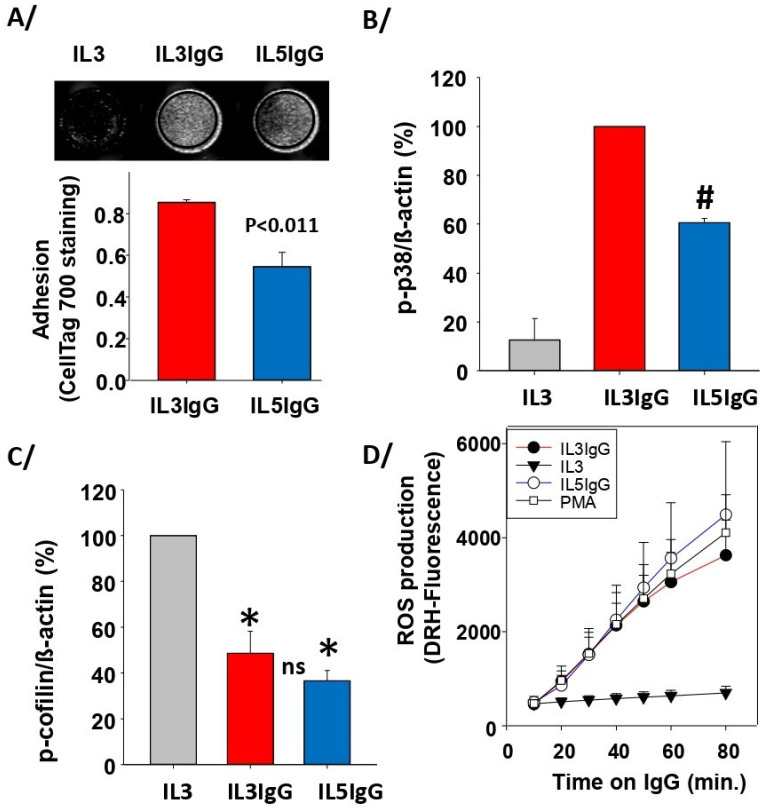
IL5-primed cells produce the same amount of ROS and phospho(p)-cofilin on IgG as IL3-primed eosinophils but display reduced adhesion and p-p38. Blood eosinophils were primed with either IL3 (2 ng/mL) or IL5 (2 ng/mL) for 20 h and were seeded on coated heat-aggregated (HA)-IgG (IL3IgG or IL5IgG) for the indicated timepoints. IL3-primed eosinophils were also seeded in wells without coated HA-IgG (IL3). (**A**) Adhesion using Cell Tag 700 was performed in 96-well plates after 2 h on IgG. Representative wells are shown in the top panel. The graph shows average ± SEM for 4 experiments using 4 different donors, and paired Student’s *t* test between IL5IgG and IL3IgG is shown. (**B**,**C**) Western blots were performed to evaluate the level of phosphorylation of p38 MAPK (**B**) and cofilin (**C**). Primed eosinophils remained 1 h (**B**) and 2 h (**C**) on IgG. The intensity of the signals is relative to β-actin, and the average ratio was fixed at 100 for IL3IgG in (**B**), and IL3 in (**C**). Average ± SD of 3 experiments with 3 different donors is shown. One-way ANOVA: IL5IgG is significantly reduced compared to IL3IgG in (**B**) (#); both IL5IgG and IL3IgG are significantly reduced compared to IL3 in (**C**) (*), with no statistical difference between IL5IgG and IL3IgG (ns). (**D**) ROS production was measured using DRH-123 at the indicated timepoints on IgG. For positive control, eosinophils were treated with PMA (100 ng/mL). No difference was observed between IL5IgG, IL3IgG, and PMA for all timepoints (average ± SD of *n* = 4 experiments with 4 different donors).

Comparing IL5 (low cytolysis) with IL3 (high cytolysis) [18], we investigated whether an early critical step, such as adhesion to IgG, was reduced after IL5 priming. Since adhesion is required for the downstream events leading to cytolysis [18], we could speculate that a significant reduction in adhesion by IL5-primed cells could explain the lack of cytolysis. Using a recently reported adhesion assay [23], which overcomes the large loss of EPX by degranulation from the IL3-primed adherent cells, we showed that IL5 led to 35% less adhesion compared to IL3 (Figure 1A). We also found a reduction in p38 mitogen-activated protein kinase (MAPK) phosphorylation in IL5-primed eosinophils (Figure 1B), but a similar dephosphorylation of cofilin compared to IL3 (Figure 1C). However, because of the slight but significant decrease in adhesion after IL5 priming compared to IL3 (Figure 1A), it was surprising to observe that ROS production on IgG was similar for both cytokines (Figure 1D).

### 3.2. Autophagic Flux Is Higher after IL5 Priming than IL3 Priming

**Figure 2:**
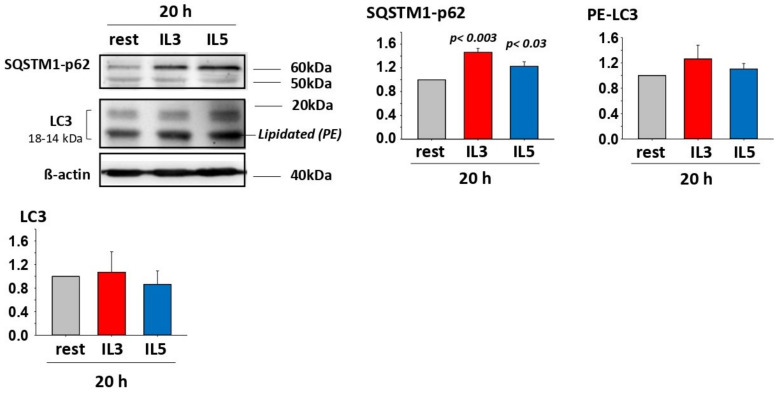
Primed eosinophils produce SQSTM1-p62, LC3 and lipidated-LC3 (PE-LC3). Blood eosinophils were cultured without cytokine (rest) or were primed with either IL3 (2 ng/mL) or IL5 (2 ng/mL) for 20 h. A representative Western blot is shown and graphs show the average ± SEM of the ratio with β-actin from 3 experiments using 3 different donors. The ratio for rest was fixed at 1. SQSTM1-p62 is increased by cytokine priming while LC3 and PE-LC3 were not changed. One-way ANOVA was performed and *p* values compared to rest for SQSTM1-p62 are shown.

**Figure 3:**
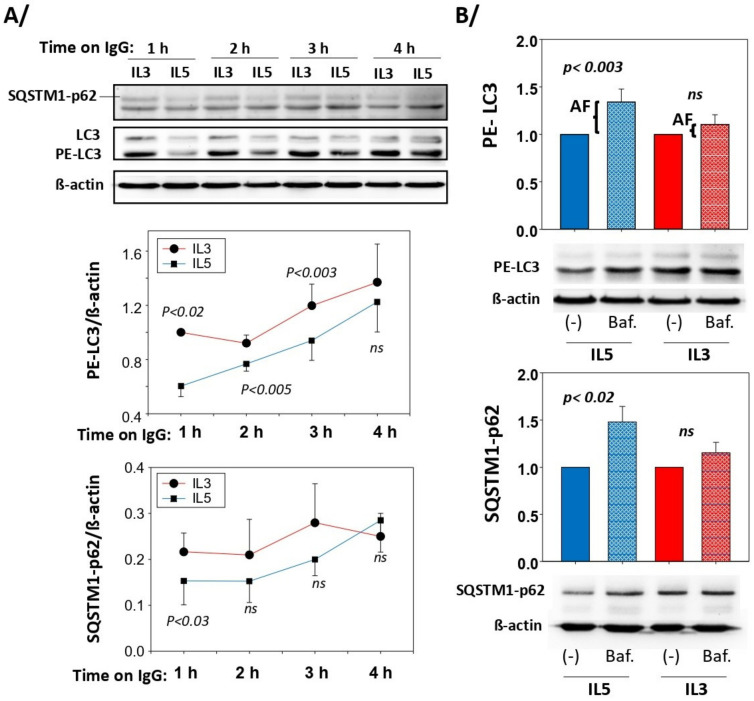
Autophagy is higher in IL5- versus IL3-primed eosinophils. Blood eosinophils were primed with IL3 (2 ng/mL) or IL5 (2 ng/mL) for 20 h and were seeded on coated HA-IgG for the indicated timepoints. Western blots for SQSTM1 and LC3 were performed and β-actin was used as a loading control. Representative blots and graphs with average ± SEM are shown. (**A**) The amount of PE-LC3 and SQSTM1-p62 was lower in IL5-primed versus IL3-primed eosinophils when interacting with IgG (*n* = 3 experiments using 3 different donors). For each timepoint, paired Student’s *t* tests to compare IL3 and IL5 were performed and *p* values < 0.05 are shown (ns = not significant). (**B**) Western blots were performed to measure PE-LC3 and SQSTM1-62 in cytokine-primed (IL3 or IL5) eosinophils on IgG for 1.5 h after treatment with bafilomycin-A1 (Baf.; 1 μM) or DMSO (vehicle only; (-)). The difference in amount of PE-LC3 with Baf. and vehicle only represent the autophagic flux (AF). Values for (-) were fixed at 1. Paired Student’s *t* tests were performed to evaluate the difference between Baf. and vehicle only (-). *P* values are shown; *n* = 6 experiments (6 subjects) for PE-LC3B and *n* = 7 (7 subjects) for SQSTM1-62.

Similar production of ROS from either IL5 or IL3 primed cells (Figure 1D) suggested that an event downstream of ROS is regulating cytolysis differentially depending on the cytokine. Autophagy is known to be activated by oxidative stress to eliminate oxidized cellular elements and thereby protects against cell death [24,25,26]. We thus analyzed events involved in autophagy. At the end of the autophagy signaling pathways, lipidated-MAP1L3CB (PE-LC3) and SQSTM1-p62 are two critical proteins implicated in the formation of autophagosomes [27,28]. Therefore, we first examined the presence of these proteins after IL3 and IL5 priming in eosinophils. Figure 2 depicts the presence of both SQSTM1-p62 and PE-LC3 in eosinophils, with a significant increase in SQSTM1-p62 as a result of both IL3 and IL5 priming. Increased autophagy is typically accompanied by a reduction in PE-LC3 and SQSTM1-p62 protein amounts due to their degradation in the autophagolysosomes [28,29,30]. We then analyzed the amount of these proteins in IL3-primed eosinophils seeded on IgG over time. We observed a small diminution of these proteins in the first 3 h on IgG versus no IgG (Supplementary Figure S1), suggesting the possible induction of autophagy in IL3-primed eosinophils on IgG. When comparing IL3 (high cytolysis) with IL5 (low cytolysis)-primed eosinophils on IgG over time, the amounts of SQSTM1-p62 and PE-LC3 were significantly lower after IL5 priming, particularly early (around 1 h) after interaction with IgG (Figure 3A), suggesting a delay in the autophagic process after IL3 priming. We then calculated the autophagic flux in both priming conditions after interaction with IgG for 1.5 h. As displayed in Figure 3B, blockade of autophagolysosome formation with bafilomycin-A1 significantly reduced the degradation of both PE-LC3 and SQSTM1-p62 in the IL5 priming condition. The increased accumulation of PE-LC3 and SQSTM1-p62 proteins by bafilomycin-A1 after IL3 priming did not reach statistical significance (Figure 3B). These data indicate higher autophagic flux in IL5-primed eosinophils.

### 3.3. Blockade of Autophagolysosome Formation Increases IL5-Primed Eosinophil Lysis on IgG

**Figure 4:**
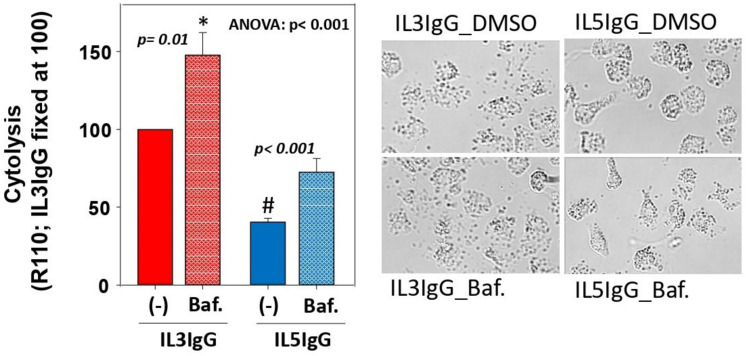
Inhibition of the formation of autophagolysosomes leads to increased eosinophil cytolysis on IgG. Eosinophils were primed with either IL3 (2 ng/mL) or IL5 (2 ng/mL) for 20 h, treated with bafilomycin-A1 (Baf.; 1μM) or vehicle only [(-); DMSO] for 20 min, and seeded on HA-IgG for 4.5 h before addition of R110 for 30 min. Cytolysis was measured by fluorescence (485 nm_Ex_/520 nm_Em_). Values obtained with the controls (medium only and IL3-primed eosinophils seeded without IgG) were subtracted from the values obtained for cytokine-primed eosinophils on IgG (IL3IgG and IL5IgG). For each of the 4 experiments (4 different blood donors), the value for IL3IgG was fixed at 100. After Log10 transformation, one-way ANOVA followed by Holm-Sidak was performed and *p* values comparing Baf. versus no Baf. are shown. # indicates *p* < 0.001 between (-)IL3IgG and (-)IL5IgG, and * indicates *p* < 0.001 between Baf.IL3IgG and Baf.IL5IgG. A representative image of the 4 conditions is shown (bright-field; 20X objective of a Nikon Eclipse Ti inverted microscope).

Next, to show the impact of autophagolysosome formation on eosinophil cytolysis, we treated IL3- and IL5-primed eosinophils with bafilomycin-A1 before interaction with IgG, and then measured cytolysis using rhodamine-110 (R110). Treatment with bafilomycin-A1 increased IL3-primed and IL5-primed eosinophil lysis by 50% and 100%, respectively (Figure 4), suggesting that autophagolysosome formation protects from cytolysis for both IL3- and IL5-primed eosinophils. A representative picture of eosinophils for each of the four conditions is shown in Figure 4. Of note, unlike bafilomycin-A1, treatment with 3-methyladenine (3-MA; 50 μg/mL; inhibitor of PI3K) did not augment cytolysis, and treatment with rapamycin (0.05 μg/mL; inhibitor of mTOR and inductor of macroautophagy) did not protect from cytolysis (Supplementary Figure S2).

### 3.4. The Formation of Autophagolysosomes Controls the Release of DNA Traps

**Figure 5:**
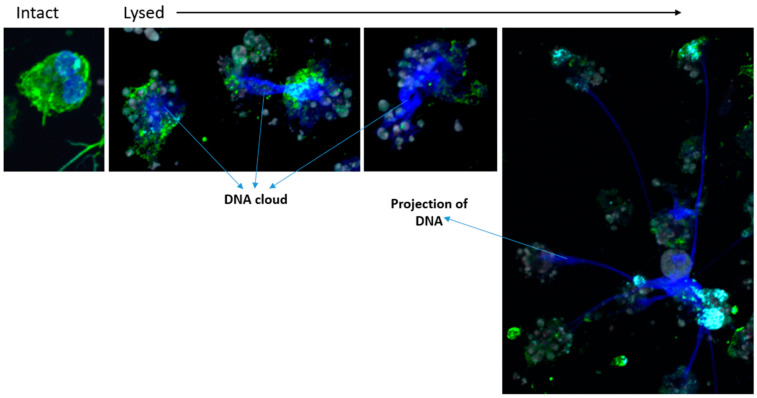
IL3-primed eosinophils on IgG release DNA in the extracellular compartment. Blood eosinophils were primed with IL3 (2 ng/mL) for 20 h and were seeded on coated HA-IgG for 5 h. Eosinophils were stained for an actin regulatory protein, gelsolin (anti-gelsolin; green), filamentous actin (phalloidin, grey), and DNA (DAPI; blue). DNA clouds and projections are stained by DAPI.

**Figure 6:**
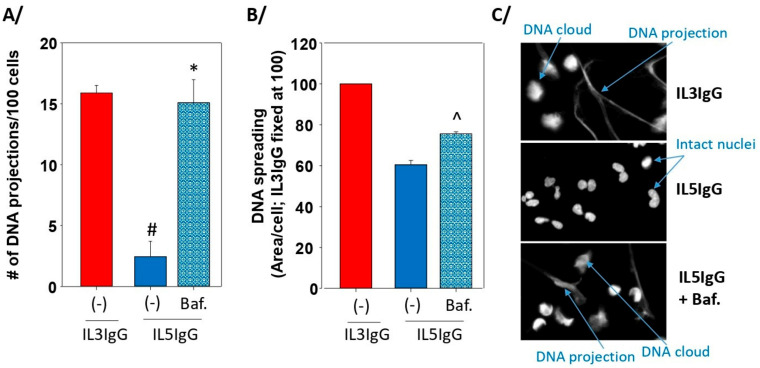
Inhibition of the formation of autophagolysosomes in IL5-primed eosinophils on IgG leads to increased DNA projections and spreading. Eosinophils were primed with IL3 (2 ng/mL) or IL5 (2 ng/mL) for 20 h and were seeded on coated HA-IgG for 4.5 h. For IL5 priming, cells were treated with bafilomycin-A1 (Baf.; 1 μM) or DMSO ((-); vehicle only) for 20 min before seeding on IgG. Cells were fixed and stained with DAPI to visualize DNA. Three experiments using three different blood donors were performed. For each experiment, three to four fields per well were imaged for a total of about 450 cells using a 20× objective. (**A**) The number of DNA projections was counted and reported per 100 cells. One-way ANOVA followed by Holm-Sidak method: * *p* < 0.001 between IL5IgG and Baf.IL5IgG; # *p* < 0.001 between (-)IL3IgG and (-)IL5IgG. (**B**) The surface covered by DNA was measured by ImageJ and reported as area of DNA per cell. IL3IgG was fixed at 100. Student’s *t* test between IL5IgG and Baf.IL5IgG: ^ *p* < 0.0014. (**C**) A representative image of the three conditions is shown.

As in other models of eosinophil cytolysis on IgG, our model also leads to rupture of the nuclei and release of DNA. Some lysed cells display spreading of DNA without long projections (DNA clouds) while other cells exhibit extended projections of DNA, typically associated with eosinophil cytoplasmic content (Figure 5). To evaluate the role of autophagolysosome formation on the rupture of the nucleus and the projection of DNA, the number of DNA projections and the surface of DNA were counted and measured, respectively in IL3-primed and IL5-primed eosinophils with or without bafilomycin-A1 treatment. Eosinophils were seeded on IgG for 4.5 h and DNA was stained with DAPI. We found that inhibition of autophagolysosome formation by bafilomycin-A1 led to a substantial increase in the number of DNA projections after IL5-priming, reaching the same count as after priming with IL3 (~15 projections per 100 cells; Figure 6A). The area of DNA (DNA clouds plus projections) per cell was also significantly enhanced by bafilomycin-A1 (Figure 6B). A representative image from the three conditions depicting intact nuclei, DNA projections, and DNA clouds is shown in Figure 6C. Of note, in a preliminary experiment, the number of DNA projections between IL3 and IL3 plus bafilomycin-A1 was very similar (~16 projections per 100 cells); thus, the IL3 plus bafilomycin-A1 condition was not repeated and is not included in Figure 6A.

## 4. Discussion

In this study, using two in vitro conditions that trigger either high (IL3) or low (IL5) levels of eosinophil cytolysis on IgG, we showed the important role of autophagolysosome formation, an end-stage critical step of autophagy, to maintain eosinophil integrity, and to avoid the release of eosinophil content and DNA traps. The protective effect of autophagy against eosinophil lysis on IgG following ROS production is in agreement with the work by Radonjic-Hoesli et al. wherein rapamycin-induced autophagy led to a reduction in eosinophil cytolysis [16].

In our previous reports [18,23], we showed that most of the IL3-primed eosinophil cytolysis starts after 3 h on IgG. Here, changes in PE-LC3 and SQSTM-p62 protein levels and increased autophagic flux in IL5-primed eosinophils occurred during the first 3 h on IgG. This indicates that autophagic flux is ongoing prior to cytolysis, and it likely protects IL5-primed eosinophils from cytolysis.

A second important observation from this work is that treatment of IL5-primed eosinophils with bafilomycin-A1 did not permit cytolysis to reach the levels observed with IL3-primed eosinophils. This suggests that other factors such as adhesion and the activation of p38 MAPK, for instance, are critical to reach a high level of cytolysis. Conversely, the release of DNA projections by bafilomycin-A- treated IL5-primed cells, reached the same level as IL3-primed eosinophils. These data are indicative of a tighter relationship between autophagolysosome formations and DNA projections more so than with cytolysis per se. The close relationship between autophagy and DNA released from eosinophils is in accordance with a study by Germic et al. showing that the absence of AGT5 (an important element of macroautophagy) is associated with increased degranulation and release of DNA from eosinophils rather than with cell survival [31].

Although the intracellular signaling between ROS production and the autophagolysosome formation was not examined in this study, the significant reduction in p38 phosphorylation in IL-5 primed versus IL-3 primed eosinophils suggests a possible role for p38 to mediate autophagy. In fact, we have previously reported that downstream of ROS production, p38 phosphorylation is required for eosinophil cytolysis on IgG [18]. In addition, it is known that p38 activation can limit autophagy activity by reducing the maturation of the autophalysosomes [32,33,34]. A fuller account of the intracellular signaling pathways involved in the modulation of autophagy in our model will be subject of further investigations.

Autophagy plays a major role in many diseases, including immune diseases [35,36], neurodegenerative diseases [37], atherosclerosis [38], and cancers [39,40]. Our present work suggests that autophagy may also have a role in eosinophilic asthma. It has been previously shown that sputum granulocytes from severe asthma subjects display a higher level of autophagy than sputum granulocytes from non-severe asthma subjects and healthy individuals [41]. However, these observations do not indicate whether autophagy is protective or damaging in asthma. The role of autophagy in vivo has been shown in an ovalbumin (OVA)-induced pulmonary inflammation mouse model using 3-MA as an inhibitor of autophagy [42]. In that study, autophagy enhanced eosinophil accumulation and DNA released from eosinophils (EEtosis) in the airways, which appears to contradict our findings. However, it is important to note that Silveira et al. found that 3-MA reduces the oxidative stress, which is an event upstream of autophagy [24,25,26]. In addition, it is well-known that 3-MA inhibits both type-III and type-I PI3K [43], the latter of which has autophagy-independent important functions, including cell adhesion and the production of ROS [16,18]. Finally, 3-MA is known to notably accelerate the spontaneous apoptosis of neutrophils [44], which could explain the reduction in eosinophilic inflammation by 3-MA. Therefore, in this in vivo model, 3-MA may have targeted other mechanisms and/or upstream events rather than eosinophil autophagy directly. In the current study, we also did not observe enhanced cytolysis by 3-MA as we did using bafilomycin-A1. While we had reported that an inhibitor of type-I PI3K strongly reduced ROS production in our model [18], we did not further examine a possible 3-MA-induced reduction in ROS production in the present study. Another study in an OVA-challenge mouse model also found beneficial outcomes to a reduction in autophagy using 3-MA and *Atg5* shRNA treatment [45]. Very recently, Daubeuf et al. used a synthetic phosphopeptide (P140) to inhibit chaperone-mediated autophagy (CMA) [30]. They found that P140 treatment reduced airway inflammation following house dust mite (HDM) sensitization and challenge. In that study, it remains unclear whether P140 affects airway inflammation via CMA and/or macroautophagy (i.e., autophagolysosome formation). Importantly also, intranasal delivery of P140 had no effect on local inflammation while intravenous delivery reduced airway inflammation (eosinophils, lymphocytes, and neutrophils), indicating a systemic role rather than a direct local function on tissue mature inflammatory cells. Further studies in animal models using tools targeting more specific autophagy signaling pathways (macroautophagy, mitophagy, microphagy, or CMA) and specific cell types (eosinophils, epithelial cells, fibroblasts, progenitors, or mature cells, etc.), would be helpful to develop therapies to control airway inflammation, eosinophil cytolysis and release of DNA traps.

Importantly, in addition to stopping the end-stage step of autophagy, bafilomycin-A1, by blocking the proton pump on the granules in eosinophils [46], may also reduce the alkalization of the cytoplasm during the oxidative burst in this model [47], and thus enhance eosinophil death and cytolysis. These events should be considered to further investigate the intracellular events leading to eosinophil cytolysis and the release of DNA.

In human asthma, we previously observed that after IL5 inhibition with mepolizumab treatment, the number of intact airway eosinophils was strongly reduced, but the airway deposition of toxic proteins (EPX) and free granules was not significantly attenuated [8]. Therefore, it is tempting to speculate that in the absence of IL5, the rate of eosinophils undergoing cytolysis is largely increased. The reduction in the release of damaging eosinophil content by augmenting airway autophagy may be of importance in eosinophilic diseases, including asthma, particularly in the absence of IL5 signaling.

## 5. Conclusions

Our study suggests that autophagy reduces mature eosinophil cytolysis and the release of their content, including DNA traps. Although in animal models of allergic inflammation it may be beneficial to inhibit autophagy to limit inflammatory cell differentiation and recruitment to the airways, enhanced autophagy locally in the airways, particularly in eosinophils, may help prevent the release of their damaging content. Studies in human models are required to measure autophagy in airway eosinophils.

## Data Availability

No data included in this manuscript have been placed in a public repository. Data can be made available upon request to the corresponding author.

## Supplementary Materials

The following supporting information can be downloaded at: https://www.mdpi.com/article/10.3390/cells11111821/s1, Figure S1. The amount of SQSTM1 and lipidated-LC3 are reduced in IL3-primed eosinophils on IgG versus no IgG; Figure S2. Cytolysis of IL 3 primed eosinophils on IgG is not increased by 3 methyladenine or decreased by rapamycin
