# Supplementary material for: Autophagy Protects against Eosinophil Cytolysis and Release of DNA
Source: Cells. 2022 Jun 2;11(11):1821. doi: 10.3390/cells11111821 (PMC9180302; doi:10.3390/cells11111821)
Supplement: Supplementary file 1 [file cells-11-01821-s001.zip › cells-1743144-supplementary.pdf]

Supplemental Figure S1

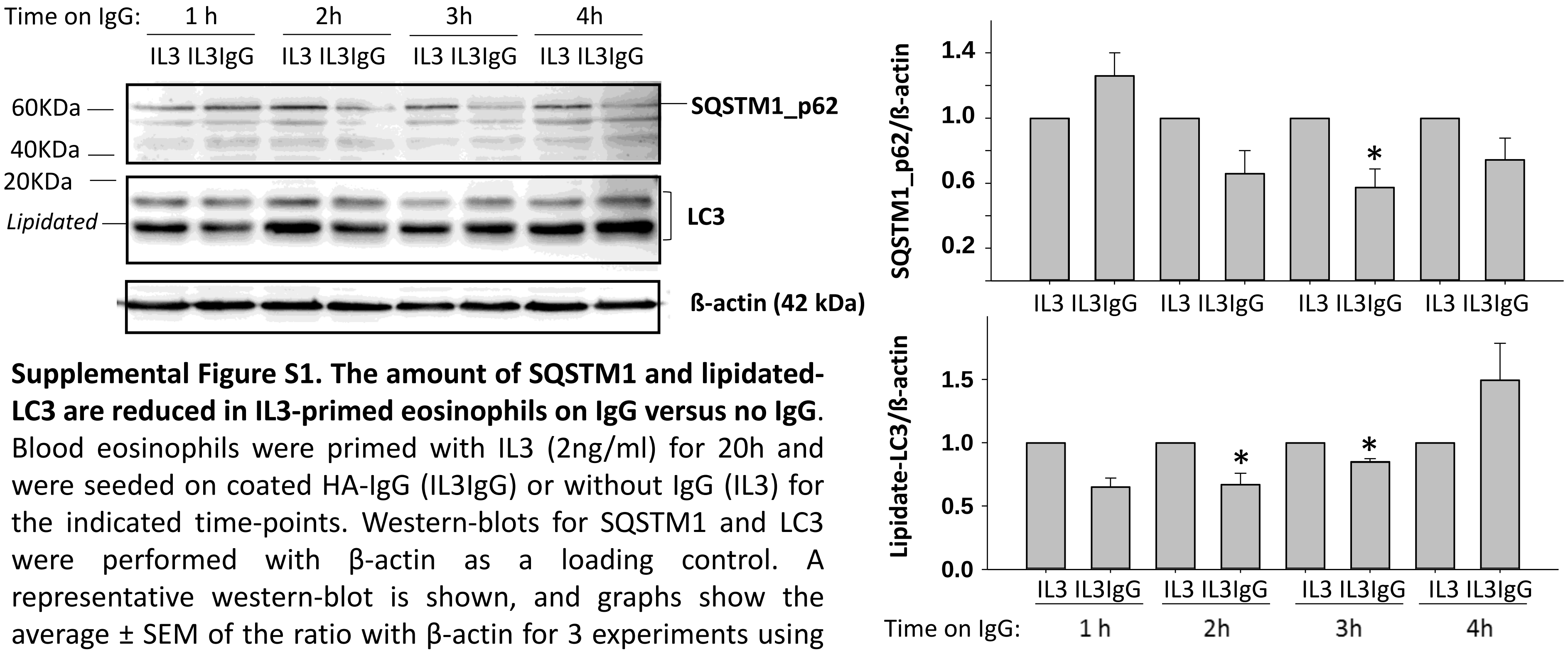

Supplemental Figure S1. The amount of SQSTM1 and lipidated-LC3 are reduced in IL3-primed eosinophils on IgG versus no IgG. Blood eosinophils were primed with IL3 (2ng/ml) for 20h and were seeded on coated HA-IgG (IL3IgG) or without IgG (IL3) for the indicated time-points. Western-blots for SQSTM1 and LC3 were performed with β-actin as a loading control. A representative western-blot is shown, and graphs show the average ± SEM of the ratio with β-actin for 3 experiments using 3 different donors (except n=2 for the 1h time-point). For each time-point, the values for IL3 were fixed at 1. Paired Student's *t* test was used to compare IL3 and IL3IgG were performed and * indicates *p* values < 0.05.

Supplemental Figure S2

<u>Inhibitors:</u>

- **Bafilomycin-A1 (BA; 1 µg/ml): Autophagolysosome formation inhibitor.**
- **3-Methyladenine (3M; 50 µg/ml): PI3K-I and III (autophagy) inhibitor**
- **Rapamycin (RA; 0.05 µg/ml): mTOR inhibitor and autophagy enhancer.**

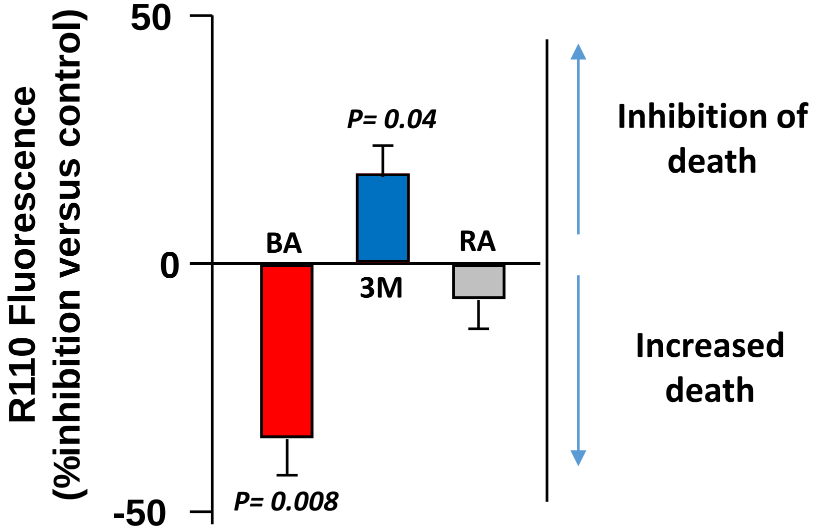

Supplemental Figure S2. Cytolysis of IL3-primed eosinophils on IgG is not increased by 3-methyladenine or decreased by rapamycin. Eosinophils were primed with IL3 (2 ng/ml) for 20 hours and were treated with the indicated inhibitors for 15 minutes before seeding on IgG for 5 h. Bis-AAF-R110 substrate was then added for 30 min, and fluorescence was measured. Data are presented as percentage (%) inhibition of cytolysis versus vehicle (mean ± SEM). *p* values are indicated on the graph (n = 3 to 4 subjects per condition).
